# Nausea and Vomiting following Balanced Xenon Anesthesia Compared to Sevoflurane: A Post-Hoc Explorative Analysis of a Randomized Controlled Trial

**DOI:** 10.1371/journal.pone.0153807

**Published:** 2016-04-25

**Authors:** Astrid V. Fahlenkamp, Christian Stoppe, Jan Cremer, Ingeborg A. Biener, Dirk Peters, Ricarda Leuchter, Albrecht Eisert, Christian C. Apfel, Rolf Rossaint, Mark Coburn

**Affiliations:** 1 Department of Anesthesiology, University Hospital Aachen, Aachen, Germany; 2 Hospital Pharmacy, University Hospital Aachen, Aachen, Germany; 3 Department of Anesthesia and Perioperative Care, University of California San Francisco, San Francisco, California, United States of America; University Hospital Oldenburg, GERMANY

## Abstract

**Objective:**

Like other inhalational anesthetics xenon seems to be associated with post-operative nausea and vomiting (PONV). We assessed nausea incidence following balanced xenon anesthesia compared to sevoflurane, and dexamethasone for its prophylaxis in a randomized controlled trial with post-hoc explorative analysis.

**Methods:**

220 subjects with elevated PONV risk (Apfel score ≥2) undergoing elective abdominal surgery were randomized to receive xenon or sevoflurane anesthesia and dexamethasone or placebo after written informed consent. 93 subjects in the xenon group and 94 subjects in the sevoflurane group completed the trial. General anesthesia was maintained with 60% xenon or 2.0% sevoflurane. Dexamethasone 4mg or placebo was administered in the first hour. Subjects were analyzed for nausea and vomiting in predefined intervals during a 24h post-anesthesia follow-up.

**Results:**

Logistic regression, controlled for dexamethasone and anesthesia/dexamethasone interaction, showed a significant risk to develop nausea following xenon anesthesia (OR 2.30, 95% CI 1.02–5.19, p = 0.044). Early-onset nausea incidence was 46% after xenon and 35% after sevoflurane anesthesia (p = 0.138). After xenon, nausea occurred significantly earlier (p = 0.014), was more frequent and rated worse in the beginning. Dexamethasone did not markedly reduce nausea occurrence in both groups. Late-onset nausea showed no considerable difference between the groups.

**Conclusion:**

In our study setting, xenon anesthesia was associated with an elevated risk to develop nausea in sensitive subjects. Dexamethasone 4mg was not effective preventing nausea in our study. Group size or dosage might have been too small, and change of statistical analysis parameters in the post-hoc evaluation might have further contributed to a limitation of our results. Further trials will be needed to address prophylaxis of xenon-induced nausea.

**Trial Registration:**

EU Clinical Trials EudraCT-2008-004132-20

ClinicalTrials.gov NCT00793663

## Introduction

PONV is regarded as any episode of nausea, vomiting, or retching, within 24h after general anesthesia [1]. Depending on the type of anesthesia its incidence can attain 20–30% in an average population [2]. Higher PONV rates have been found in sensitive subjects [3]. PONV can be distinguished between an early phase in the first 2h after anesthesia, and a late phase between 2 and 24h following anesthesia [2]. PONV affects individual comfort [4] and might even influence the surgical outcome [5]. Despite individually predisposing factors, the use of volatile and gaseous anesthetics has been identified as one of the main causes for PONV in sensitive subjects [6–8]. Several strategies graded according to individual risk have been evaluated for PONV prevention and treatment [9–12].

The noble gas xenon is an anesthetic with many advantageous effects [13], e.g. its hemodynamic stability [14–16] and its experimental neuroprotection [17]. Xenon is an antagonist at the 5- HT3-receptor and thus might exert antiemetic properties [18]. A recent observational study found a lower rate of PONV than predicted by individual Apfel score following xenon anesthesia in a mixed patient population [19]. However, randomized clinical studies have shown a higher PONV incidence after xenon anesthesia compared to propofol [20].

The primary aim of this study was to investigate the development of nausea following xenon anesthesia in PONV sensitive subjects and compare it to the volatile anesthetic sevoflurane. The secondary aim was to further estimate the efficacy of dexamethasone as a preventive prophylactic to reduce xenon-induced nausea.

## Materials and Methods

### Study Design and Ethics

The study was designed and performed as a mono-center, factorial, randomized controlled clinical trial according to the Declaration of Helsinki. The factorial design consisted of several distinct study arms [21–23], of which the occurrence and prophylaxis of nausea following xenon anesthesia is subjected here. The study subjects were blinded to the randomized anesthetic, and the prophylactic antiemetic treatment was administered in a double-blinded, placebo-controlled manner.

This study was designed to test nausea incidence following volatile anesthesia in PONV-sensitive subjects (Apfel score ≥ 2). Thus, the design inherited a possibly higher risk for participants to develop nausea and/or vomiting. All subjects were explicitly informed about this possibly elevated risk of nausea and/or vomiting during their study participation before obtaining written informed consent. Furthermore, it was determined in the study protocol that randomized study subjects who dropped out of the study procedure were not replaced to avoid any possible risk of repeating cases with severe nausea. Ethical approval for this study was provided by the ethics committee of the Medical Faculty of the University Hospital RWTH Aachen in Germany on 24 October 2008 (Chairperson: Professor G. Schmalzing, MD; reference number: EK 110/08). This design was furthermore approved by the German official authority for supervision and approval of pharmaceuticals (Bundesinstitut fuer Arzneimittel und Medizinprodukte). The trial was registered at the EMA (EudraCT No.: 2008-004132-20) and at ClinicalTrials.gov (NCT No.: 00793663) (http://clinicaltrials.gov).

### Subjects

Enrolment was performed between November 2008 and March 2011. Study physicians screened all patients scheduled for elective abdominal surgery (i.e. gynecological, urological or abdominal surgery, with either open or laparoscopic approach) with a planned duration ≥ 60 min and a planned admission to the ward after post anesthesia care unit (PACU) stay. This procedure led to a screening rate of approx. 5 patients per day and an approx. total of 3000 patients over the recruitment period. A screening log was not implemented. After written informed consent, 220 patients aged 18–75 years, ASA status I-II and Apfel’s simplified PONV predicting score of 2–4 (i.e. a PONV likelihood of about 40–80%) [3;24] were enrolled in the trial. Enrolment was performed by the study physicians. Among exclusion criteria were severe cardiac, respiratory, liver or kidney function disorders, known or suspected allergic reaction to one of the study medications, suspicion of malignant hyperthermia, pregnancy/lactation period, legal incapacity to give informed consent and refusal to participate.

### Randomization

Two independent permuted-block randomizations were performed; the first for type of anesthesia (Xenon vs. Sevoflurane; block size 10; allocation ratio 1:1), and the second for type of prophylactic (dexamethasone vs. placebo; block size 10; allocation ratio 1:1). The randomization lists were generated by computer allocation using a randomization-software (RandList version 1.2, DatInf) by a member of the clinic staff independent from the study team and sealed in an opaque envelope and locked in an office cupboard only accessible for the head investigator in case of emergency unblinding due to a serious adverse event. Randomization codes for the type of anesthesia were sealed in opaque envelopes parameterized with the corresponding study number on the outside, and were opened by the study physician on the day of surgery shortly before the start of intervention. Anesthesia technique was blinded to the participants throughout their trial participation. Randomization codes for the type of prophylactic treatment were consigned to the hospital pharmacy, who received an email with the according study number on the eve of surgery for each individual participant. The hospital pharmacy then provided a blinded syringe containing the randomized prophylactic treatment in equal amount of liquid, marked with the individual study number. Participants, study physicians, and outcome assessors were blinded to the type of prophylactic treatment.

### Trial Procedure

**Fig 1:**
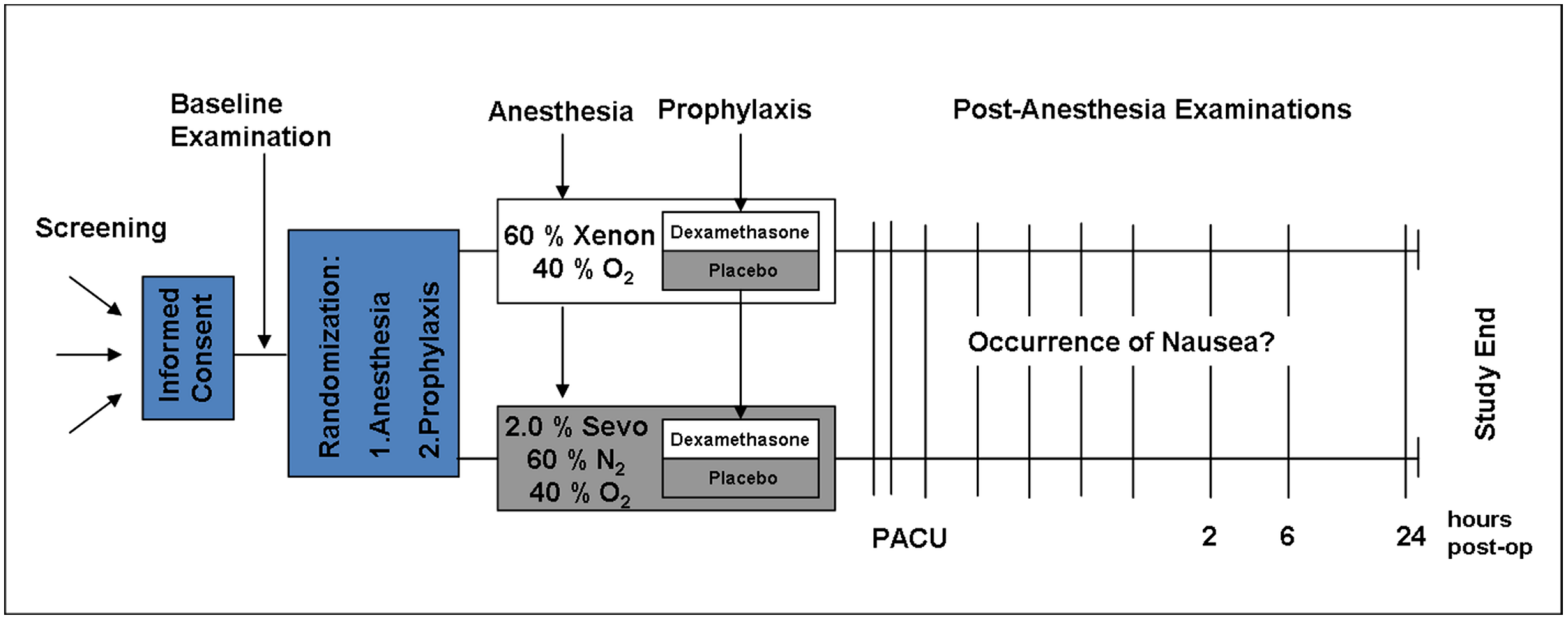
Study procedure. A simplified sketch of the study procedure is shown in Fig 1. After obtaining written informed consent and baseline examination, patients were first randomized to receive xenon or sevoflurane anesthesia for their individually scheduled surgery, then randomized to receive dexamethasone or placebo for prophylaxis of post-operative nausea. Within the first hour of anesthesia, the randomized prophylactic treatment was applied. Patients were monitored for 24h following anesthesia in fixed intervals (during PACU stay at 0, 5, 15, 30, 45, 60, 75, 105, 120 min until discharge as well as 2h, 6h, and 24h post anesthesia). The study-specific survey was completed 24h post anesthesia.

A sketch of the study procedure is displayed in Fig 1. Following premedication with oral midazolam 7.5 mg 45 min before induction, general anesthesia was induced by a bolus of propofol (2.0 mg^1^kg^-1^ initially, repeating dose if considered necessary 0.5–1.0 mg^1^kg^-1^) and 0.5 μg^1^kg^-1^min^-1^ remifentanil infusion for 60s. Endotracheal intubation was facilitated by 0.6 mg^1^kg^-1^ rocuronium. Xenon or sevoflurane wash-in was started with a target end-tidal concentration of 60±5 vol.% xenon or 2±0.2 vol.% sevoflurane in min. 30% oxygen. Within the first hour patients received a single injection of randomized prophylactic treatment (dexamethasone 4 mg or placebo NaCl 0.9%, provided by the hospital pharmacy in a blinded syringe, same liquid volume with the concordant study number). General anesthesia was maintained through inhalation of xenon or sevoflurane and supported by remifentanil infusion titrated to clinical needs. Standard monitoring included pulse oximetry, three channel ECG, non-invasive arterial blood pressure measurement, control of the tube cuff pressure, neuromuscular monitoring, core temperature, assessment of end-tidal oxygen, end-tidal carbon dioxide and end-tidal anesthetic gas concentration. Vital parameters (i.e. heart rate, systolic and diastolic blood pressure (AS/3 monitor, GE Datex-Ohmeda, Helsinki, Finland), end-tidal O_2_, CO_2_) were automatically recorded throughout anesthesia at 10s intervals and logged every five min. End-tidal concentrations of anesthetics and infusion rate of remifentanil were logged at the same intervals. Depth of anesthesia was assessed by standard physiological criteria [21]. Normoxia, a physiological carbon dioxide concentration and normal body temperature were maintained. Medical quality xenon in steel cylinders was provided by Air Liquide Santé International (Paris, France). Sevoflurane was provided by Abbott (Wiesbaden, Germany). Both anesthetic agents were administered using a closed circuit respirator (Felix Dual^®^, Taema, France) with concordant software allowing the use of xenon only under closed circuit conditions [25].

Standard treatment of blood loss, fluid replacement and hemodynamic support were applied when indicated. Twenty minutes ahead of the estimated end of surgery piritramide 0.05 mg^1^kg^-1^, and either paracetamol 10 mg^1^kg^-1^ or metamizole 15 mg^1^kg^-1^ were administered for post-anesthetic pain management. Remifentanil was stopped when all painful interventions were terminated. Anesthesia was discontinued by washing out of the inhalational anesthetic via high-flow 100% oxygen when all surgical interventions including bandaging were terminated and complete recovery from neuromuscular block was assured. Extubation was accomplished when patients’ upper airway reflexes were recovered, breathing was regular and sufficient (breathing frequency >8 min^-1^; breathing volume >6 ml^1^kg^-1^), and patients were able to react on demand (eye opening, swallowing, breathing). Patients were observed in the operation room until full recovery, and then transported to the post anesthetic care unit (PACU) for further observation. Pain medication (piritramide 0.02–0.05 mg^1^kg^-1^) was titrated to clinical needs.

Nausea was assessed asking subjects about their individual nausea levels rated with an eleven-point numeric rating scale (NRS) with ‘0’ indicating no nausea and ‘10’ indicating worst nausea ever experienced. Patients were measured at fixed time-points upon admission to the PACU, after five and fifteen minutes and thereafter in fifteen-minute intervals. The primary outcome parameter was the development of subjective nausea, defined as NRS ≥ 2, at any time-point in the 0-2h PACU interval. Episodes of vomiting were recorded at the same time points.

Follow-up examinations were performed at 6h, and 24h after anesthesia and patients were questioned about the incidence and their specific levels of nausea during these time periods (2-6h and 6-24h post anesthesia) with an eleven-point NRS as described above. Occurrence of vomiting and number of vomiting episodes were assessed in the same intervals. At the end of their study participation (24h post anesthesia) patients were asked to rate the individual quality of their anesthesia with a six-point rating scale according to the German school grade system, with 1 as best and 6 as worst.

### Statistics

The primary outcome parameter was the development of early-onset nausea defined as NRS ≥ 2 at any time-point in the 0-2h PACU interval. The parameter nausea was selected as the most sensitive (in general nausea as a symptom of PONV appears first) and most reliable (subjects do not vomit without nausea) determinant characterizing PONV. Secondary outcome parameters were the occurrence and intensity of early-onset nausea, and the influence of dexamethasone on early onset nausea. The incidence of vomiting during the first two hours following anesthesia and the occurrence of late-onset nausea and vomiting during the 24h post-anesthesia survey were evaluated as well.

The *a priory* calculation of the quantity of subjects and subsequent power of the trial was performed for the total of several independent study arms as described in the study design section [21–23], using nQuery Advisor^®^ Version 7.0 (Statistical Solutions, Saugus, Massachusetts, USA). Based on an expected mean Apfel score of 3, a PONV -and thereby nausea- incidence of about 60% in the sevoflurane group and a similar incidence in the xenon group were hypothesized. A reduction of nausea by dexamethasone of about a third (i.e. from 60% to 40%) was assumed to be relevant. The *a priory* power was calculated for the H0 hypothesis that dexamethasone prophylaxis did not cause a different level of reduction of nausea with xenon anesthesia compared to sevoflurane anesthesia. With an alpha error of 0.05 (i.e.) and a beta error of 0.2 (i.e. 80% power), we calculated a need of 107 patients per group. A total of 110 patients per group was planned for inclusion to account for drop-outs.

When planning for the trial and *a priory* calculation were implemented, we didn’t take into account that there might be a difference in nausea incidence between xenon and sevoflurane anesthesia, which was noted when the statistical analysis was performed. Due to this important observation, a high drop-out rate entailing a lower participant number for analysis than *a priory* expected plus the fact that the trial protocol was lacking a total statistical analysis plan, we decided to change the analysis parameters and conduct a *post-hoc* analysis with the primary outcome parameter incidence of post-operative nausea defined as above and the H0 hypothesis that xenon anesthesia had no different incidence of nausea than sevoflurane anesthesia. We performed an intention to treat-analysis, including those patients who had completed the randomized anesthesia and had withdrawn preterm because of severe nausea, but did not account for the dropped-out participants due to other reasons (see Fig 1) that had not completed the randomized anesthesia (i.e. no implementation of missing values to obtain the full analysis set). The primary outcome parameter was evaluated with a risk analysis via binary logistic regression (influence of xenon, dexamethasone, and the combination of dexamethasone and xenon on the risk to develop significant nausea), followed by a direct comparison via one-way ANOVA. The secondary outcome parameters, including the isolated incidences of significant nausea following xenon and sevoflurane anesthesia, were directly compared with bivariate analyses. Logistic regression was performed as the primary efficacy analysis; all further p-values were understood merely descriptive. Statistical analysis was performed using IBM SPSS statistics software version 20.0 (SPSS Inc., Chicago, Illinois, USA). Figures were generated with GraphPad PRISM^®^ (GraphPad Software Inc., La Jolla, California, USA).

## Results

**Fig 2:**
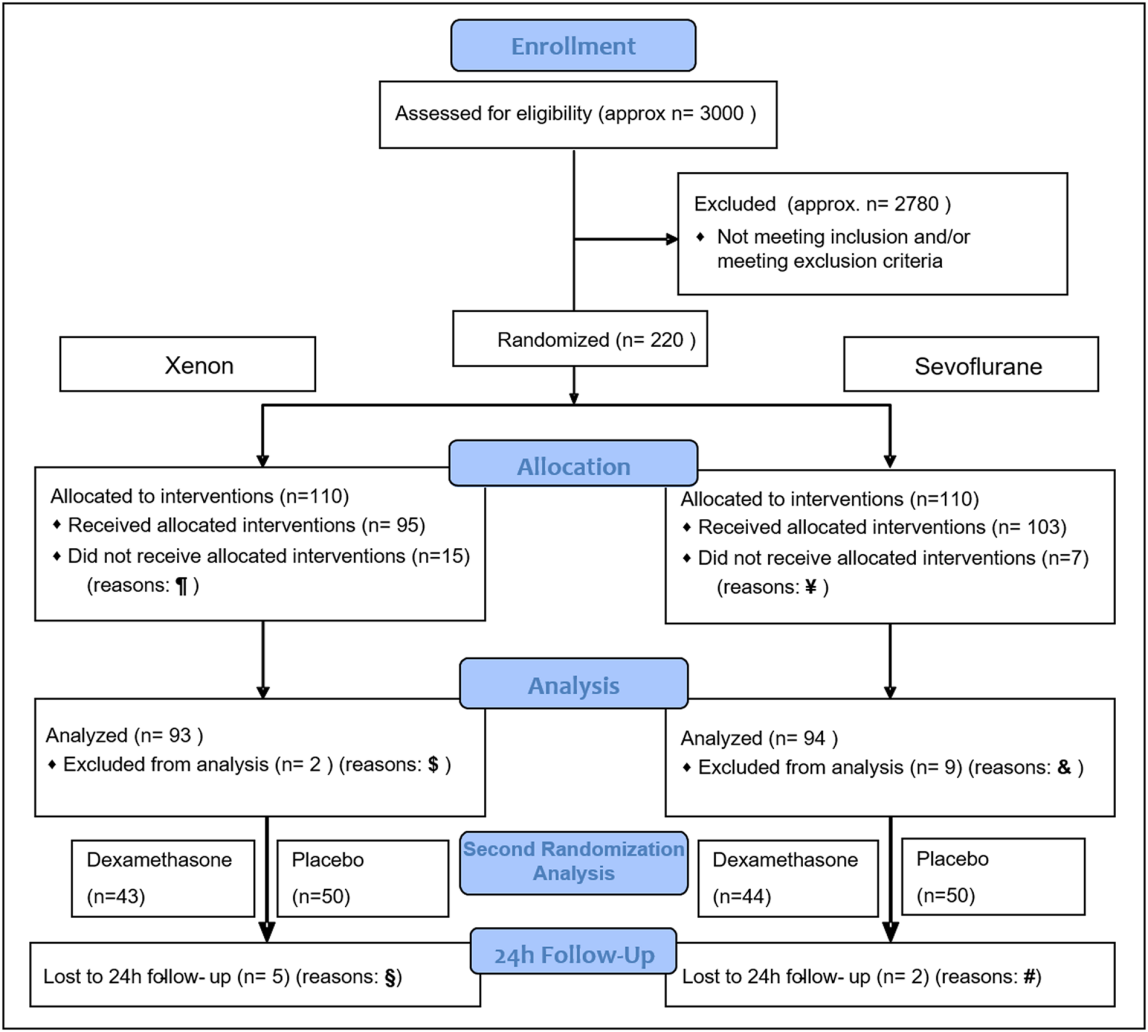
Consort flow chart. A detailed flow chart depicts all patients enrolled in the trial including the reasons for study drop-out. 220 patients in total were randomized after written informed consent into one of the same-sized study groups. Pre-interventional drop out in the xenon group occurred in fifteen cases (¶): Five patients withdrew their consent; five were excluded after randomization for safety reasons. Two patients did not receive the scheduled surgery; three did not receive the allocated intervention for administrative reasons. Post-interventional drop-out in the xenon group occurred in two cases ($) by exclusion from data analysis due to study protocol violation. 93 patients in the xenon group received the allocated intervention and data analysis. Of these patients, 43 received dexamethasone as randomized prophylactic treatment, whereas 50 received placebo. Five patients (§) withdrew preterm because of severe nausea. Their data until the time of dropout was included into the final analysis. Pre-interventional drop-out in the sevoflurane group occurred in seven cases (Ұ): One patient withdrew his consent; two were excluded after randomization for safety reasons. Two patients did not receive the scheduled surgery; two did not receive the allocated intervention for administrative reasons. Post interventional drop-out in the sevoflurane group occurred in nine cases (&): One patient dropped out due to a serious adverse event not associated with the study intervention. Eight patients of the sevoflurane group were excluded from data analysis because of violation of the study protocol. 94 patients in the sevoflurane group received the allocated intervention and data analysis, with 44 cases of dexamethasone as randomized prophylactic treatment and 50 placebo. Two patients (#) did not complete the 24h-follow-up because of withdrawal due to severe nausea. Their data until the time of dropout was included into the final analysis.

A detailed flow chart of patient inclusion, randomization and study flow including reasons for drop-out is presented in Fig 2. 93 patients in the xenon group and 94 patients in the sevoflurane group received the allocated anesthesia and interventions plus admission for data analysis. 88 subjects following xenon anesthesia and 92 subjects following sevoflurane anesthesia completed the 24h follow-up. The groups were not different with respect to patient age, height, body mass index, gender, ASA status, PONV history and Apfel Score (Table 1). The mean Apfel Score was lower than expected (Table 1).

**Table 1:** Baseline patient characteristics.

Group	Xenon (n = 93)	Sevoflurane (n = 94)
Age [yrs]	48.0 ± 14.8	49.3 ± 14.1
Height [m]	1.72 ± 0.1	1.70 ± 0.1
BMI [kg m^-2^]	26.2 ± 4.8	25.1 ± 4.7
Sex m/f [n] (%)	39/54 (42/58)	31/63 (33/67)
ASA I/II/III [n] (%)	46/46/1 (49.5/49.5/1)	43/50/1 (46/53/1)
History of PONV y/n [n] (%)	10/83 (11/89)	12/82 (13/87)
Apfel Score 2/3/4 [n] (median)	54/33/6 (2)	56/32/6 (2)

A demographic baseline description of the subjects randomized for xenon or sevoflurane anesthesia are presented in Table 1. Age, height, and body mass index (BMI) are given as mean ± SD. Gender (m male/f female), ASA classification and history of PONV (y yes/n no) are shown in numbers and percent of total. The individual Apfel score is given in numbers by scoring and group median.

The study groups did not differ with respect to type of surgery, anesthesia duration, additional epidural, and ongoing and post anesthesia piritramide doses (Table 2). Relevant differences were observed in the total dose of propofol administered for induction, and the use of oxygen and remifentanil during maintenance of anesthesia (Table 2). The PACU stay tended to be longer in the xenon group.

**Table 2:** Key data of the study intervention.

Group	Xenon (n = 93)	Sevoflurane (n = 94)	P
Surgery visc/gyn/uro [n] (%)	6/50/37 (6/54/40)	3/60/31 (3/64/33)	0.296
Epidural none/lumb/thor [n] (%)	68/4/21 (73/4/23)	73/2/19 (78/2/20)	0.625
Anesthesia duration [min]	141.7 ± 60.3	145.3 ± 62.3	0.688
PACU stay [min]	94.6 ± 56.9	80.4 ± 40.4	0.050
∑ Propofol [mg kg^-1^]	3.0 ± 1.0	2.3 ± 0.6	0.000
Ø FiO_2_	0.44 ± 0.06	0.51 ± 0.12	0.000
Ø FiAnesthetic [%] (MAC)	56 ± 5 (0.9)	1.8 ± 0.4 (1.0)	-
Ø Remifentanil [μg kg^-1^ min^-1^]	0.19 ± 0.06	0.18 ± 0.06	0.046
Ø Piritramide (An) [mg kg^-1^]	0.08 ± 0.05	0.07 ± 0.04	0.178
Ø Piritramide (PACU) [mg kg^-1^]	0.08 ± 0.08	0.09 ± 0.08	0.580

Key data of the study intervention are demonstrated in Table 2. The length of anesthesia and PACU stay, the total amount (Σ) of propofol, the average values (Ψ) of inspiratory oxygen fraction (FiO_2_) and anesthetic concentration (FiAnesthetic), the average remifentanil and piritramide doses administered during general anesthesia (An) and during PACU stay are presented as mean ± SD. The FiAnesthetic as a fraction of its individual minimal alveolar concentration (MAC) is presented in parentheses. The surgery type (visc visceral/ gyn gynecological/ uro urological) and an optional additional epidural anesthesia (none/ lumb lumbar/ thor thoracic) are given as numbers and percent of total.

**Fig 3:**
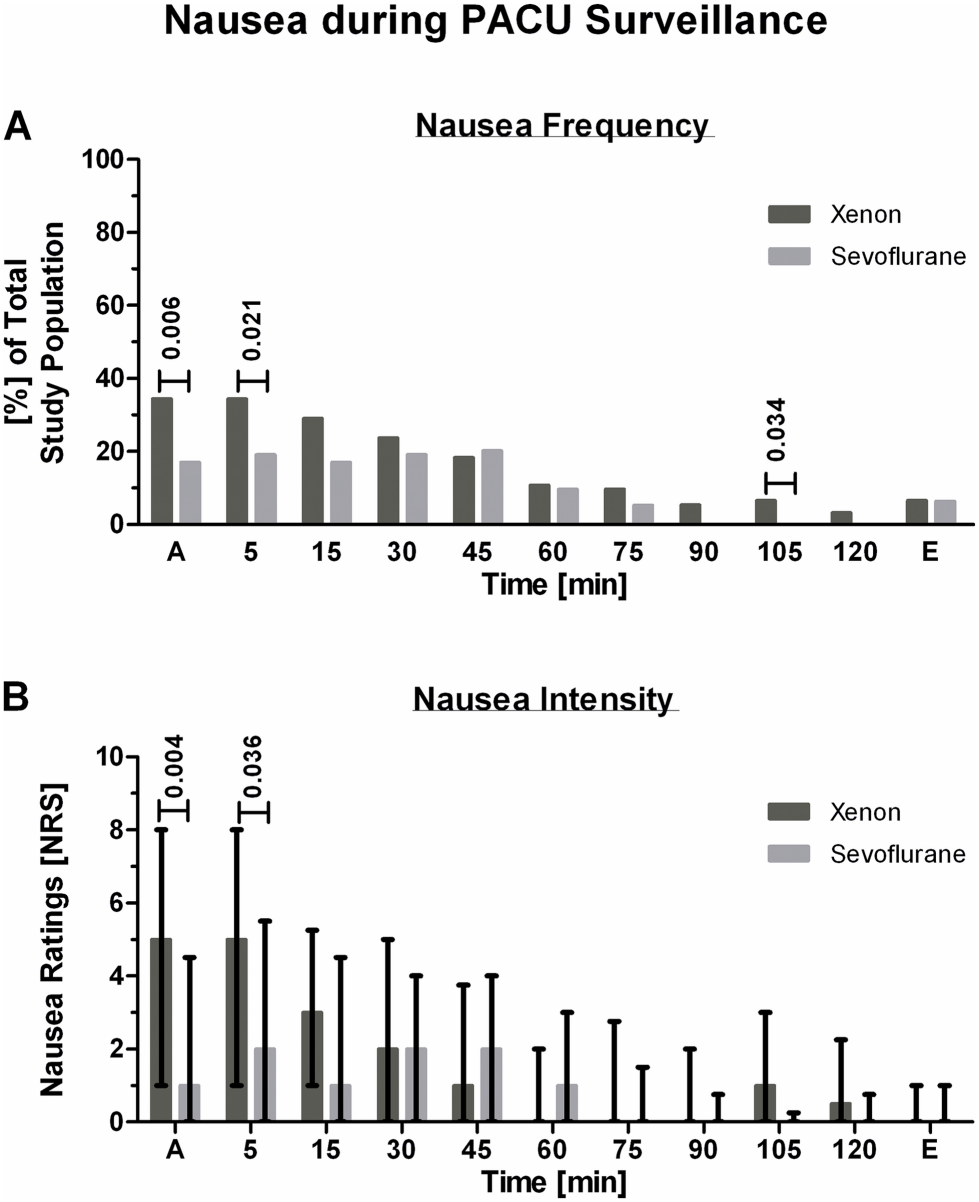
Significant nausea during PACU surveillance. The timed occurrence of nausea experienced by study participants during their PACU stay is depicted in Fig 3. Fig 3A shows the frequency of significant nausea in study participants, given as percent of total. The nausea intensity ratings of subjects experiencing significant nausea are presented in Fig 3B as medians and quartiles.

Binary logistic regression analysis controlled for dexamethasone and the interaction of anesthesia and dexamethasone revealed an elevated risk to develop post-operative nausea following xenon anesthesia (OR 2.302, 95% CI 1.021–5.190, p = 0.044). Application of dexamethasone and the combination of xenon anesthesia and dexamethasone did not significantly influence the risk to develop post-operative nausea (OR 1.338, 95% CI 0.572–3.128, p = 0.502 and OR 0.451, 95% CI 0.138–1.475, p = 0.188, respectively). The isolated incidences (bivariate analysis) of early-onset nausea and vomiting or retching following xenon or sevoflurane anesthesia did not statistically differ (Table 3). The time until patients first asked for further antiemetic medication was significantly shorter in the xenon group (Table 3). The occurrence of significant nausea was significantly more frequent following xenon anesthesia during the first 5 min and after 105 min of PACU stay (Fig 3A). Individual nausea ratings of subjects experiencing nausea were higher following xenon anesthesia during the first 5 min of PACU stay (Fig 3B). Five patients after xenon and two patients after sevoflurane anesthesia withdrew preterm because of severe nausea (Fig 2).

**Table 3:** Nausea and vomiting following xenon or sevoflurane anesthesia.

**Time**	**Early-onset nausea and vomiting**	**Xenon (n = 93)**	**Sevoflurane (n = 94)**	**P**
0-2h	Nausea [n] (%)	43 (46.2)	33 (35.1)	0.138
0-2h	Vomiting/Retching [n] (%)	16 (17.2)	13 (13.8)	0.551
0-2h	Time to application of antiemetic medication [min]	8.0 ± 14.0	18.4 ± 21.6	0.014
**Time**	**Late-onset nausea and vomiting**	**Xenon (n = 88)**	**Sevoflurane (n = 92)**	**P**
2-6h	Nausea [n] (%)	25 (28.4)	21 (22.8)	0.399
2-6h	Vomiting/Retching [n] (%)	16 (18.2)	15 (16.3)	0.844
6-24h	Nausea [n] (%)	18 (20.5)	13 (14.1)	0.324
6-24h	Vomiting/Retching [n] (%)	5 (5.7)	6 (6.5)	1.000

Nausea and vomiting following xenon and sevoflurane anesthesia are presented in Table 3. Results are distinguished in early-onset (0-2h post anesthesia) and late-onset nausea and vomiting (2-6h and 6-24h post anesthesia).

Late-onset nausea (2-6h and 6-24h post anesthesia) and vomiting were not affected by anesthetic technique in a relevant way (Table 3).

**Fig 4:**
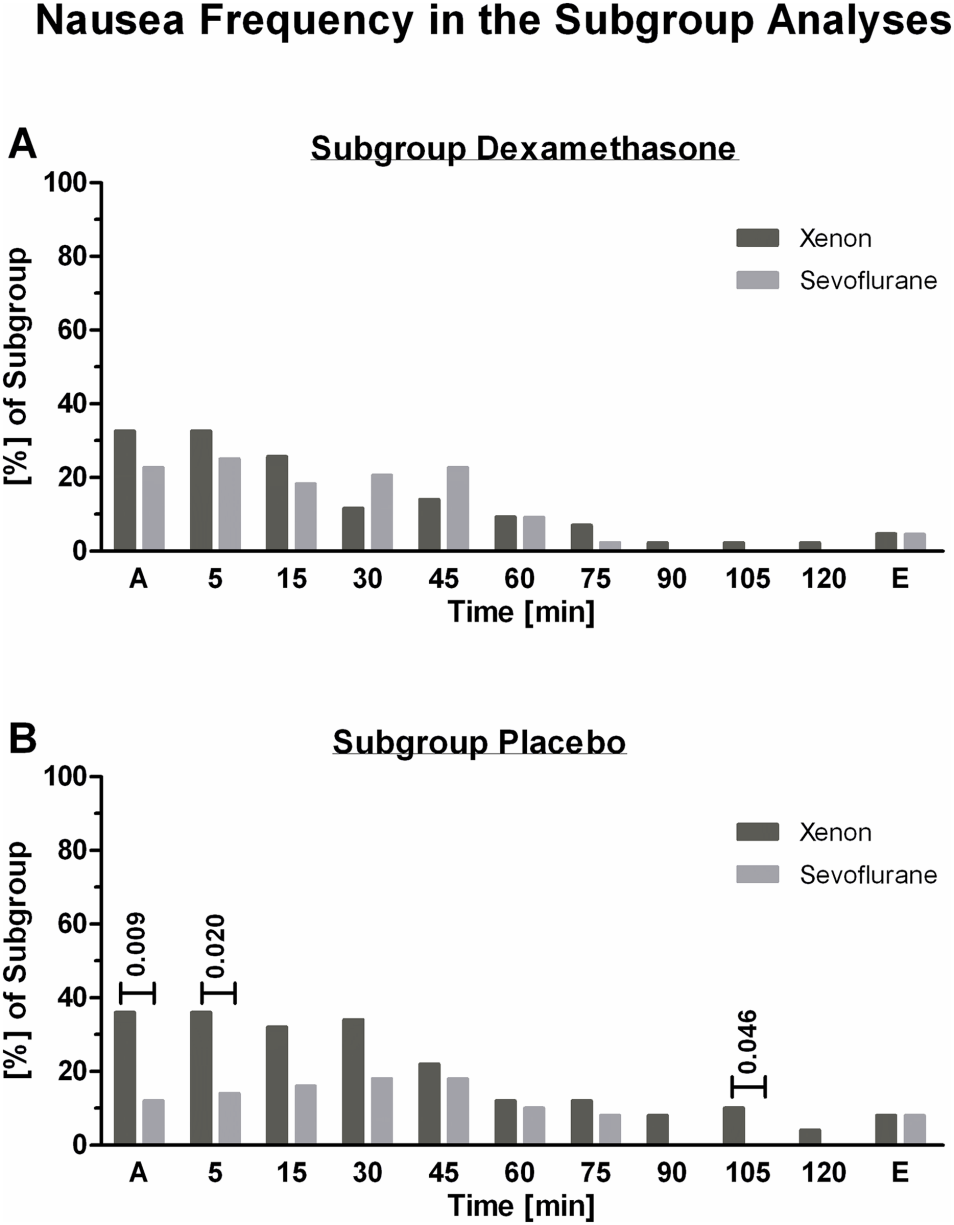
Frequency of nausea in the dexamethasone and placebo subgroups. The timed frequency of significant nausea experienced by study participants during their PACU stay differentiated by randomized prophylactic is shown in Fig 4. Fig 4A shows nausea frequency in study participants following dexamethasone prophylaxis; Fig 4B depicts nausea frequency following placebo. All values are given as percent of subgroup and randomized anesthesia, accordingly.

The overall incidences of early-onset nausea and of vomiting did not differ in the subgroup analysis of dexamethasone for prophylaxis in comparison to placebo following xenon or sevoflurane anesthesia (Table 4). Regarding the timed occurrence of significant nausea, frequencies of nausea following dexamethasone prophylaxis did not differ between xenon or sevoflurane anesthesia (Fig 4A). Frequencies of nausea following placebo application showed a statistical difference during the first 5 min of PACU stay, with higher values following xenon anesthesia (Fig 4B).

**Table 4:** Subgroup analysis of dexamethasone prophylaxis on nausea and vomiting following xenon or sevoflurane anesthesia.

		*Xenon*		*Sevoflurane*	
**Time**	**Early-onset nausea and vomiting**	**Dexa. (n = 43)**	**Placebo (n = 50)**	**Dexa. (n = 44)**	**Placebo (n = 50)**
0-2h	Nausea [n] (%)	17 (39.5)	26 (52.0)	17 (38.6)	16 (32.0)
0-2h	Vomiting/Retching [n] (%)	5 (11.6)	11 (22.0)	3 (6.8)	10 (20.0)
0-2h	Time to application of antiemetic medication [min]	10.3 ± 18.9	6.2 ± 9.1	14.5 ± 17.2	22.3 ± 25.2
**Time**	**Late-onset nausea and vomiting**	**Dexa. (n = 42)**	**Placebo (n = 46)**	**Dexa. (n = 43)**	**Placebo (n = 49)**
2-6h	Nausea [n] (%)	10 (23.8)	15 (32.6)	6 (14.0)	15 (30.6)
2-6h	Vomiting/Retching [n] (%)	5 (11.9)	11 (23.9)	5 (11.6)	10 (20.4)
6-24h	Nausea [n] (%)	6 (14.3)	12 (26.1)	6 (14.0)	7 (14.3)
6-24h	Vomiting/Retching [n] (%)	2 (4.9)	3 (6.5)	3 (7.0)	3 (6.1)

Nausea and vomiting following xenon and sevoflurane anesthesia in the subgroup analyses of xenon respective sevoflurane anesthesia and dexamethasone vs. placebo prophylaxis are presented in Table 4. Results are distinguished in early-onset (0-2h post anesthesia) and late-onset nausea and vomiting (2-6h and 6-24h post anesthesia).

The incidences of late-onset nausea and of vomiting were not markedly altered following dexamethasone prophylaxis compared to placebo in the xenon group, as were the incidences following sevoflurane anesthesia (Table 4).

A relevant difference was noted between the subjective evaluation ratings for anesthesia in the 24h follow-up examination: subjective xenon anesthesia quality was rated poorer (median 2, range 1–6) than sevoflurane anesthesia (median 2, range 1–4; p = 0.006).

## Discussion

Our analyses revealed an elevated risk to develop early nausea in sensitive subjects treated with xenon. However, the overall incidences of nausea following xenon or sevoflurane anesthesia were not significantly different in this study. Nausea developed earlier and was rated worse in the xenon group. Late-onset nausea was not markedly different in both groups.

Xenon anesthesia reveals a higher incidence of PONV compared to propofol anesthesia [20]. Of note, so far PONV rates after xenon anesthesia compared to other anesthesia techniques have only been extrapolated from secondary data or risk analyses in observational studies [13;19]. The findings of aforementioned studies range from a higher expectancy [13] to a lower probability to develop PONV [19] when exposed to xenon. Our study is the first that directly compares nausea rates after xenon to a volatile anesthetic. While all results have to be interpreted with care, we do draw the conclusion that xenon anesthesia is associated with a higher likelihood to develop nausea than volatile anesthesia. Regression analyses showed an elevated risk to develop early-onset nausea. Nausea was significantly more frequent in the xenon group shortly after emergence of anesthesia. The lack of significance in overall nausea incidences in our study is difficult to interpret. One possible explanation might be a partly similarity of action of the two compared anesthetics. Like xenon, the reference anesthetic sevoflurane is an antagonist at the 5-HT3-receptor and might act more like xenon than like other volatile anesthetics when examining nausea [18]. Another possibility might be a different action of dexamethasone in the study groups. In bivariate analysis, nausea incidences in the xenon and sevoflurane group were calculated including both dexamethasone and placebo cases. In logistic regression analysis the influence of xenon anesthesia on nausea rates was calculated independent of dexamethasone. When looking at the subgroup analyses nausea rates differed in numbers between xenon/dexamethasone and xenon/placebo, while rates in the sevoflurane subgroups hardly differed. This observation is unusual, since dexamethasone has been shown to be effective in the prevention of PONV following sevoflurane anesthesia [26;27]; but it probably accounts for the differences observed here. Results would assumedly have been more distinct with a higher number of study participants. Due to a high drop-out rate and other factors listed in the limitations section, we did not achieve the initially planned number of participants. However, the *a priory* power calculation that was base to the planned subject number is not applicable to the results of this study as the originally expected Apfel score and estimated PONV incidence of the calculation were not attained, and the statistical analysis was performed *post-hoc*.

In total, nausea occurred earlier, and the initial nausea ratings were higher following xenon anesthesia. One plausible explanation may be the fact that patients after xenon anesthesia emerged faster and independent of anesthesia duration [28–30], and thus may experience an uncomfortable physical state earlier and more vividly. In addition, the fast washout of xenon might also account for a faster termination of 5-HT3-antagonism, leaving space for various counteracting, pro-emetic stimuli. A bias through a misdistribution of well-established PONV enhancing risk factors—like female gender, history of PONV, age, amount of post-operative opioids, duration of anesthesia [3;6] is minimized since these factors were evaluated and are equally distributed between the groups and subgroups. However further possible influencing circumstances, like fasting, surgery type, hormonal status, or further factors were not or only partially logged and evaluated in our study. Yet, due to scant evidence aforementioned risk factors have lately been discouraged from their use for PONV risk assessment [6]. Of note, the incidence of nausea in the xenon group in our study met the expected PONV rate according to the median Apfel score of 2 (46% vs. approximately 40% expected) [3]. We did not find a lower PONV rate than expected in the xenon group as described in a recently published work that analyzed risk factors predicting PONV in a large unselected population [19]. However, there were several significant differences between the named study and our work which might have accounted for the different findings: Schaefer and colleagues did not limit their inclusion criteria to an Apfel score ≥ 2. Thus they included quite a large number of participants being Apfel score 0 or 1. Moreover, this study identified female sex and long duration of anesthesia as two of the most important risk factors inducing PONV. We randomized more than 60% females and the average duration of anesthesia was more than 140 min in both groups. This might have contributed to the higher nausea rate in our study.

Volatile anesthetics have mainly been held responsible to develop early PONV within the first two hours after the end of anesthesia. However, volatile anesthetics seem not responsible for late PONV occurring in the first 24 h post anesthesia [7]. This seems to be the same for xenon. Nausea rates and episodes of vomiting following xenon anesthesia showed a similar decline towards 24h as compared to the sevoflurane group. However, these results may be biased by the less frequent examinations during the later postoperative period. Subjects might simply have superseded further episodes of nausea and/or vomiting.

The evaluation of anesthesia was worse when xenon had been administered. This result seems congruent with the higher risk to develop nausea following xenon anesthesia, and should entail future strategies to reduce this risk and improve individual well-being. The faster emergence and high alertness following xenon anesthesia [28;29] enables patients to remember their PACU stay and their post-operative body state in detail. In consequence this might also contribute to the subjective evaluations. Fast recovery from anesthesia is a highly favorable attribute vis à vis anesthetists, surgeons, and hospital economists. However, it might not prove to be optimal for the individual patient.

Effective prophylactic strategies have been evaluated for the prevention of PONV to abate this annoying side effect after general anesthesia. Dexamethasone is known to be preventive for PONV in volatile anesthetics [10;31;32]. Our results do not seem to confirm these data for xenon anesthesia. The reduction of nausea was not significantly different to the placebo group. Of note, in the sevoflurane group prophylactic dexamethasone did not lead to a reduction in nausea rates compared to placebo. Moreover, the rates of early-onset nausea following sevoflurane/dexamethasone and sevoflurane/placebo were almost identical. This finding is contradictory to previous studies [26;27]. Two possible reasons for the failure of dexamethasone might be a different dosage and time of application. In former studies dexamethasone was most effective when administered in doses of 8–10 mg and before induction of anesthesia [33;34], two conditions that were not accomplished during our trial. However, a recent meta-analysis showed that a 4 mg dose of dexamethasone seems to have a similar antiemetic efficacy to the 8–10 mg dose [10]. Another factor for the lack of effectiveness of dexamethasone prophylaxis in this trial may have been a decreased number of examined patients due to one of the various reasons, see limitations. Further studies examining therapeutic options of PONV following xenon anesthesia would thus be desirable.

### Limitations

Several factors in the design and realization of this study might limit our findings. We designed this study as a multifactorial study with several study arms which assessed independent questions within one setting. Even if independency of the study arms were ensured, there might have been hidden interactions or influences that have biased our study observations. Additionally, due to the non-replacement of subjects in case of drop-out (see methods‘ section, study design) and a high study drop-out rate due to other reasons (see Fig 1) we did not achieve the initially estimated patient number. This might have further influenced our obtained results, especially in the case of the secondary analysis performed for dexamethasone prophylaxis. However, the median Apfel score achieved in this study was 2 and thereby lower than presupposed for the calculated number of participants. In consequence this fact subsequently led to a lower nausea rate in the study population than would have been eligible especially for secondary analyses.

The statistical planning of the study also holds several limitations: The *a priory* calculation didn’t take into account the observed difference in nausea incidence between xenon and sevoflurane anesthesia. Since this difference entailed an impact on the primary endpoint definition and thus had a direct influence on all further estimations, a change in statistical analysis from the primarily planned calculation towards a post-hoc evaluation was necessary. The post hoc analysis within this existing sample might have led to a bigger type one error than expected just by looking at the p-value. Post-hoc power calculations are only marginally able to prevent this bias. Moreover, the study is likely to be underpowered with respect to at least one of the study arms in this linked design. A further lack of total statistical analysis plan in the design and the exclusion of missing values handling in the protocol and thereby loss of the full analysis set for statistical calculation have further weakening influences on the conclusions form our results. An interim analysis of the primary outcome parameter would have been very helpful to detect the tendencies concerning different incidences of nausea in both study groups, but was unfortunately neither planned nor performed for the primary endpoint initially planned for this study arm.

Within our study set we had a trend towards failure to receive allocated interventions (see Fig 1), with a maximum in the xenon group. Since reasons for this failure were seemingly manifold and incoherent (withdrawn consent, safety reasons), a missing at random assumption and treatment of missing values might have helped to identify further factors that might have strengthened the outcome of the statistical analysis. However, the handling of missing values was neither planned a priory nor implemented into the post-hoc analysis.

## Conclusion

In our post-hoc analysis xenon anesthesia is associated with an overall elevated risk to induce nausea in sensitive subjects. However early and late nausea and vomiting incidences did not statistically differ after xenon or sevoflurane in our study. Nausea was more frequent and appeared earlier and was rated worse in the xenon group. Change of statistical analysis parameters in the post-hoc evaluation might have contributed to a limitation of our results. Additional studies will be needed to address the question of prophylaxis and treatment of PONV following xenon anesthesia.

## Supporting Information

S1 File The trial protocol is available within the supplementary material. (PDF)

S2 File The Consort checklist is available within the supplementary material. (PDF)
