# Supplementary material for: Nausea and Vomiting following Balanced Xenon Anesthesia Compared to Sevoflurane: A Post-Hoc Explorative Analysis of a Randomized Controlled Trial
Source: PLoS One. 2016 Apr 25;11(4):e0153807. doi: 10.1371/journal.pone.0153807 (PMC4844115; doi:10.1371/journal.pone.0153807)
Supplement: S1 File — (PDF) [file pone.0153807.s001.pdf]

Clinical Study Protocol

Protocol title:

1) The effect of xenon and sevoflurane on depth of hypnosis monitors when titrated to standard anesthesia parameter.
2) The efficacy of dexamethasone for prevention of PONV after xenon or sevoflurane.
3) Ondansetron as rescue for the treatment of established PONV after xenon or sevoflurane.

EudraCT-number:

2008-004132-20

Protocol identifying number:

ALS-8-08-A-401

Protocol version:

Version V3; Date: 20.10.2008

Synopsis

1 GENERAL INFORMATION 5

1.1 Protocol title, protocol identifying number, version, and date. 5

1.2 Name and address of the sponsor and monitor. 5

1.3 Name and title of persons authorized to sign the protocol and the protocol amendments for the sponsor. 5

1.4 Name, title, address, and telephone number of the sponsor´s medical expert for the trial. 5

1.5 Name and title of the investigator who is responsible for conducting the trial, and the address and telephone number of the trial site. 6

1.6 Name, title, address, and telephone number of the qualified physician, who is responsible for all trial-site related medical decisions. 6

1.7 Name and address of the clinical laboratory and other medical and/or technical department and/or institutions involved in the trial. 6

2 BACKGROUND INFORMATION 6

2.1 Name and description of the investigational products. 6

2.2 A summary of findings from nonclinical studies that potentially have clinical significance and from clinical trials that is relevant to the trial. 7

2.3 Summary of the known and potential risks and benefits, if any, to the human subjects. Risk-benefit-analysis, study rationale. 7

2.4 Description of and justification for the route of administration, dosage, dosage regimen, and treatment period. 8

2.4.1 Volatile Anesthetics 8

2.4.2 Dexamethasone 8

2.4.3 Ondansetron 8

2.5 A statement that the trial will be conducted in compliance with the protocol, GCP and the applicable regulatory requirements. 8

2.6 Description of the population to be studied. 9

2.7 References to literature and data that are relevant to the trial, and that provide background for the trial. 9

3 TRIAL OBJECTIVES AND PURPOSE 10

3.1 Trial objectives: Number and issue of the study arms, if several objectives are investigated. 10

3.2 Trial purpose. 10

4 TRIAL DESIGN 10

4.1 A specific statement of the primary endpoints and the secondary endpoints, if any, to be measured during the trial. 10

4.1.1 Primary endpoints: 10

4.1.2 Key secondary endpoint(s): 10
4.2 A description of type/design of trial to be conducted. 11
4.3 A description of measures taken to minimize/avoid bias, including: 11
4.4 A description of the trial treatments and the dosage and dosage regimen of the investigational products. Also include a description of the dosage form, packaging, and labelling of the investigational products........ 11
4.5 The expected duration of the subject participation, and a description of the sequence and duration of all trial periods, including follow-up, if any. 12
4.6 A description of 'stopping rules' or 'discontinuation criteria' for individual subjects, parts of the trial and entire trial. 12
4.7 Accountability procedures for the investigational products, including the placebos........ 12
4.8 Maintenance of the trial treatment randomization codes and procedures for breaking codes. .. 13
4.9 The identification of any data to be recorded directly on the CRFs, and to be considered to be source data 13
5 SELECTION AND WITHDRAL OF SUBJECTS 14
5.1 Subject inclusion criteria. 14
5.2 Subject exclusion criteria........ 14
5.2.1 Contraindications and safety measures as prescribed by product information sheets........ 14
5.2.2 Further exclusion criteria 14
5.3 Subject withdrawal criteria and procedures specifying: 14
5.3.1 The following withdrawal criteria are stringent to the investigational subjects 15
5.3.2 When and how to withdraw subjects from the trial/ investigational product treatment. 15
5.3.3 The type and timing of the data to be collected for withdrawn subjects. 15
5.3.4 Whether and how subjects are to be replaced. 15
5.3.5 The follow-up for subjects withdrawn from investigational product treatment/trial treatment. 15
6 TREATMENT OF SUBJECTS 16
6.1 The treatments to be administered, including the names of all products, the doses, the dosing schedules, the routes/modes of administration, and the treatment periods, including the follow-up periods for each investigational product treatment/ trial treatment group/ arm of trial........ 16
6.2 Medication/treatment permitted and not permitted before and/or during the trial. 16
6.3 Procedures for monitoring subject compliance. 17
7 ASSESSMENT OF EFFICACY 17
7.1 Specification of the efficacy parameters. 17
7.2 Methods and timing for assessing, recording, and analyzing of efficacy parameters. 17
8 ASSESSMENT OF SAFETY 17

8.1 Specification of safety parameters. 17
8.2 The methods and timing for assessing, recording, and analyzing safety parameters. 17
8.3 Procedures for eliciting reports of and for recording and reporting adverse event and intercurrent illnesses. 17
8.4 The type and duration of the follow-up of subjects after adverse events. 18
9 STATISTICS 18
9.1 A description of the methods to be employed, including timing of any planned interim analysis. 18
9.2 The number of subjects planned to be enrolled. Reason for choice of sample size, including reflections on the power of the trial and clinical justification. 18
9.3 The level of significance to be used. 19
9.4 Criteria for the termination of the trial. 19
9.5 Procedure for accounting for missing, unused, and spurious data. 19
9.6 Procedures for reporting any deviations from the original statistical plan. 19
9.7 The selection of subjects to be included in the analyses. 19
10 DIRECT ACCESS TO SOURCE DATA/DOCUMENTS 19
10.1 Sponsor Surveillance 19
10.2 Independent Superior Surveillance 19
10.3 Reporting of Trial Results 19
11 QUALITY CONTROL AND QUALITY ASSURANCE 19
11.1 Quality control 19
11.2 Quality assurance 20
12 ETHICS 20
13 DATA HANDLING AND RECORD KEEPING 20
14 FINANCING AND INSURANCE 20
14.1 Financing of the trial. 20
14.2 Study Budget 21
14.2 Insurance of the trial. 21
15 PUBLICATION POLICY 21
16 SIGNATURES 22
17 ANNEX 22
17.1 Table of Abbreviations 22
17.2 Additional Information 23

1 GENERAL INFORMATION

1.1 Protocol title, protocol identifying number, version, and date.

This protocol describes a total of three studies that are intrinsically linked. It does therefore have three titles:

1) The effect of xenon and sevoflurane on depth of hypnosis monitors when titrated to standard anesthesia parameter.

2) The efficacy of dexamethasone for prevention of PONV after xenon or sevoflurane.

3) Ondansetron as rescue for the treatment of established PONV after xenon or sevoflurane.

ALS-8-08-A-401, Version: V3; Date: 20.10.08

1.2 Name and address of the sponsor and monitor.

Air Liquide Santé International
Dr. Catherine Billoet
75 Quai d´ Orsay
F-75007 Paris
+33 (1) 40628566
catherine.billoet@airliquide.com

1.3 Name and title of persons authorized to sign the protocol and the protocol amendments for the sponsor.

Dr. med. Mark Coburn
Department of Anesthesia
University Hospital Aachen
Pauwelsstr. 30
52074 Aachen
+49 (241) 8035394
mcoburn@ukaachen.de

Univ.-Prof. Dr. med. Rolf Rossaint
Head of the Department
Department of Anesthesia
University Hospital Aachen
Pauwelsstr. 30
52074 Aachen
+49 (241) 8088179
rossaint@post.rwth-aachen.de

1.4 Name, title, address, and telephone number of the sponsor´s medical expert for the trial.

Prof. Dr. Christian C. Apfel
Perioperative Clinical Research Core
University of California San Francisco
UCSF Medical Center at Mt Zion
1600 Divisadero, C-447
San Francisco, CA 94115
+1 (415) 885 7842

apfelc@anesthesia.ucsf.edu

1.5 Name and title of the investigator who is responsible for conducting the trial, and the address and telephone number of the trial site.

Dr. med. Mark Coburn
Department of Anesthesia
University Hospital Aachen
Pauwelsstr. 30
52074 Aachen
+49 (241) 8035394
mcoburn@ukaachen.de

1.6 Name, title, address, and telephone number of the qualified physician, who is responsible for all trial-site related medical decisions.

Dr. med. Mark Coburn
Department of Anesthesia
University Hospital Aachen
Pauwelsstr. 30
52074 Aachen
+49 (241) 8035394
mcoburn@ukaachen.de

Univ.-Prof. Dr. med. Rolf Rossaint
Head of the Department
Department of Anesthesia
University Hospital Aachen
Pauwelsstr. 30
52074 Aachen
+49 (241) 8088179
rossaint@post.rwth-aachen.de

1.7 Name and address of the clinical laboratory and other medical and/or technical department and/or institutions involved in the trial.

Central Pharmacy
University Hospital Aachen
Head of the Department
Dr. rer. nat. Albrecht Eisert
Steinbergweg 20
52074 Aachen

2 BACKGROUND INFORMATION

2.1 Name and description of the investigational products.

Xenon: inhalative anesthetic
Sevoflurane: inhalative anesthetic
Dexamethasone: intravenous medication for postoperative nausea and vomiting
Ondansetron: intravenous rescue medication for postoperative nausea and vomiting

2.2 A summary of findings from nonclinical studies that potentially have clinical significance and from clinical trials that is relevant to the trial.

See 2.3

2.3 Summary of the known and potential risks and benefits, if any, to the human subjects. Risk-benefit-analysis, study rationale.

The aim of this study is ad 1) to visualize and compare the effects of xenon or sevoflurane on the depth of hypnosis as assessed by EEG state and latency of auditory evoked potentials, quantified and converted into the comparable indices BIS and cAAI. Ad 2) it is to show whether dexamethasone has a significant effect on the prevention of post operative nausea and vomiting after sevoflurane or xenon anesthesia – a purpose which it has long been used for in everyday clinical practice. Ad 3) it is to answer the question, if ondansetron, another strongly antiemetic remedy, reduces PONV during the first 32 minutes after application, and if how its kinetics proceed after sevoflurane or xenon anesthesia. The current clinical practice lacks evidence in all three cases.

Ad 1) Basis for this trial are study subjects under general anesthesia whose EEG state and AEP are noninvasively monitored. As the monitoring is noninvasive, blinded to the anesthetist, and merely descriptive – i.e. it will not be used for the regulation of depth of hypnosis and the amount of applied anesthetic drugs – the only 'risk' for the subject investigated is general anesthesia itself; a risk the subject bears the moment he/she agrees to have the proposed operation done under general anesthesia. The drugs used for general anesthesia in this trial have been authorized for use in Germany and are everyday's good anesthetic practice. The monitoring of depth of hypnosis with BIS and AEP has not yet been described for sevoflurane or xenon in detail. The estimated benefit of this trial objective is to measure the depth of hypnosis as assessed by BIS and cAAI during an average general anesthesia with xenon or sevoflurane for abdominal and gynecological surgery, and therefore to establish a reliable monitoring system for measuring and documenting the actual depth of hypnosis under general anesthesia with xenon or sevoflurane.

Ad 2) Several reviews recommend an antiemetic prophylaxis in general, if there is an increased risk for postoperative nausea and vomiting. It is good clinical practice to apply a medical prophylaxis against PONV when the Apfel score meets 2 or more points. This proceeding, however, is not implied in the German *Leitlinien* (guidelines) and is not considered as 'standard of care'. Additionally, until now no data exist that proves the effectiveness neither of dexamethasone in the case of xenon or sevoflurane anesthesia, nor of the preventive proceeding. Recent data suggest on the contrary that a prophylactic treatment is not superior to an early rescue treatment. Often the prophylactic treatment increases consumption of antiemetic medication which is not necessarily needed. However the subjects included in this trial might or might not have been applied the dose of dexamethasone to prevent PONV, so there is no additional risk expected when given the drug. A supposable risk of the investigational subjects who receive placebo is to develop PONV. PONV is a rather uncomfortable but little hazardous complication to the subjects. The trial proceeding is conducted with the patients' informed consent; and if PONV occurs, the study design implies the application of a second medication. The benefit of this trial objective is to answer the question whether 4 mg dexamethasone i.v. is an effective prophylactic treatment against PONV after xenon or sevoflurane anesthesia. This can be only addressed placebo-controlled.

Ad 3) In case of nausea occurring after general anesthesia, which usually develops with latency to the end of anesthesia during recovery in the PACU, the patient will be immediately applied a second treatment – in this case either a single dose of 4 mg

ondansetron or placebo. There is no evidence whether ondansetron is an effective remedy in case of PONV after xenon or sevoflurane anesthesia, and the benefit of this study is to acquire data to answer this question. Ondansetron has little undesirable side-effects, but unnecessary costs if ineffective should be considered. The possible risk for investigational subjects is nausea and development of emesis. As mentioned above, investigational subjects are considered healthy by inclusion criteria (chapter 5.1), so the aggravation of PONV is rather uncomfortable but little hazardous. Furthermore, the observational period is limited to 32 min after application. This is a time slot often tolerated in clinical practice for an antiemetic drug to show its effect until the next dose or drug is applied, so the applied proceeding by the trial protocol does not markedly differ from everyday clinical practice. After this period the investigational subject suffering from PONV is provided to receive any antiemetic treatment needed. Since nausea is a very subjective symptom the blinded placebo control design is necessary to gain evidence. The investigational subject is only exposed to a short temporary discomfort which he /she has agreed on in informed consent and from which the patient can be withdrawn at any time. The anticipated benefit is to gain evidence about the effectiveness and kinetics of ondansetron as antiemetic remedy after xenon or sevoflurane anesthesia.

2.4 Description of and justification for the route of administration, dosage, dosage regimen, and treatment period.

2.4.1 Volatile Anesthetics

Route of administration: inhalative (solely accredited application form)
Dosage: 0.7 – 1.0 MAC (MAC according to the age of the person investigated and the volatile anesthetic used)
Dosage regimen: continuous application adapted to actual surgical stimulus
Treatment period: duration of general anesthesia required for surgical procedure

2.4.2 Dexamethasone

Route of administration: intra venous (recommended way of application for prophylaxis and/or treatment of PONV)
Dosage: 4 mg (equal to 0.05 – 0.1mg/kg of an average adult; expert recommendation)
Dosage regimen: i.v. bolus
Treatment period: single application after the induction of general anesthesia

2.4.3 Ondansetron

Route of administration: intra venous (recommended application form for the treatment of PONV
Dosage: 4 mg (recommended dose for the treatment of PONV)
Dosage regimen: i.v. bolus
Treatment period: single dose application after the occurrence of nausea experienced by the study subject

2.5 A statement that the trial will be conducted in compliance with the protocol, GCP and the applicable regulatory requirements.

The study will be conducted in compliance with the protocol, the Note for Guidance on Good Clinical Practice (CPMP/ICH/135/95) and the applicable regulatory requirements.

2.6 Description of the population to be studied.

Study subjects will be recruited pre-operative during their in hospital stay. Inclusion criteria are: Patients ≥ 18 < 75 years; ASA physical status I-II; Planned duration of anesthesia > 60 minutes; Apfel score ≥ 2-3; Elective (laparoscopic) surgery (abdominal, gynaecological) and women: with a highly effective contraception, defined as methods with a pearl index <1 (i.e. hormonal contraceptives, IUD).

2.7 References to literature and data that are relevant to the trial, and that provide background for the trial.

1. Apfel C, Kranke P, Piper S et al. Nausea and vomiting in the postoperative phase. Expert- and evidence-based recommendations for prophylaxis and therapy. Anaesthesist 2007; 56: 1170-80.
2. Apfel C, Stoecklein K, Lipfert P. PONV: a problem of inhalational anaesthesia? Best Pract Res Clin Anaesthesiol. 2005; 19: 485-500.
3. Gan TJ, MeyerT, Apfel C, et al. Consensus guidelines for managing postoperative nausea and vomiting. Anesth Analg. 2003: 62-71.
4. Apfel C, Korttila K, Abdalla M et al. A factorial trial of six interventions for the prevention of postoperative nausea and vomiting. N Engl J Med 2004; 350: 2441-51
5. Scuderi P, James R, Harris L et al. Antiemetic prophylaxis does not improve outcomes after outpatient surgery when compared to symptomatic treatment. Anesthesiology 1999; 90: 360-71.
6. Rossaint R, Reyle-Hahn M, Baumert J, et al. Multicenter randomized comparison of the efficacy and safety of xenon and isoflurane in patients undergoing elective surgery. Anesthesiology 2003; 98: 6-13
7. Wappler F, Rossaint R, Baumert J, et al. Multicenter randomized comparison of xenon and isoflurane on left ventricular function in patients undergoing elective surgery. Anesthesiology 2007; 106: 463-71
8. Coburn M, Kunitz O, Apfel C, et al. Incidence of postoperative nausea and emetic episodes after xenon anaesthesia compared with propofol-based anaesthesia. Br J Anaesth 2008; 100: 787-91.
9. Baumert J, Hein M, Hecker K, et al. Autonomic cardiac control with xenon anaethesia in patients at cardiac risk. Br J Anaesth 2007: 98:722-7.
10. Coburn M, Kunitz O, Baumert J, et al. Randomized controlled trial of the haemodynamic and recovery effects of xenon or propofol anaesthesia. Br J Anaesth 2005; 94: 198-202.
11. Nakata Y, Goto T, Ishiguro Y, et al. Minimum alveolar concentration (MAC) of xenon with sevoflurane in humans. Anesthesiology 2001; 94: 611-4.
12. Nickalls R, Mapleson W. Age-related iso-MAC charts for isoflurane, sevoflurane and desflurane in man. Br J Anaesth 2003; 91: 170-4.
13. Goto T, Nakata Y. Saito H, et al. The midlatency auditory evoked potentials predict responsiveness to verbal commands in patients emerging from anesthesia with xenon, isoflurane, and sevoflurane but not with nitrous oxide. Anesthesiology 2001; 94: 782-9
14. Goto T, Nakata Y, Saito H, et al. Bispectral analysis of the electroencephalogram does not predict responsiveness to verbal command in patients emerging from xenon anaesthesia. Br J Anaesth 2000; 85: 359-63
15. Punjasawadwong Y, Boonjeungmonkol N, Phongchiewboon A. Bispectral index for improving anaesthetic delivery and postoperative recovery. Cochrane Database Syst Rev 2007: 17: CD003843.
16. Schmidt G, Müller J, Bischoff P. Messung der Narkosetiefe. Anaesthesist 2008; 57: 9-36.

17. Jensen E, Litvan H, Revuelta M, et al. Cerebral state index during propofol anesthesia. Anesthesiology 2006; 105: 28-36.
18. White P, Ma H, Tang J, et al. Does the use of electroencephalographic bispectral index or auditory evoked potential index monitoring facilitate recovery after desflurane anesthesia in the ambulatory setting? Anesthesiology 2004; 100: 811-7.

3 TRIAL OBJECTIVES AND PURPOSE

3.1 Trial objectives: Number and issue of the study arms, if several objectives are investigated.

In this trial three objectives will be investigated which are intrinsically linked.
1. To compare the effects of xenon or sevoflurane on the depth of hypnosis as assessed by the Bispectral Index (BIS) and the Composite Auditory Evoked Potential Index (cAAI) when titrated according to standard clinical parameter.
2. To demonstrate that dexamethasone prevents PONV equally well after xenon or sevoflurane.
3. To determine the onset-time of ondansetron when used as rescue medication for postoperative nausea and vomiting.

3.2 Trial purpose.

The purpose of this study is ad 1) to measure the depth of hypnosis as assessed by BIS and cAAI during an average general anesthesia with xenon or sevoflurane and to establish a reliable monitoring system for measuring and documenting the actual depth of hypnosis for the volatile anesthetics investigated. Ad 2) the question is to be answered whether 4 mg dexamethasone i.v. is an effective prophylactic treatment against PONV in case of xenon or sevoflurane anesthesia. Ad 3) it serves to gain evidence about the (non-)effectiveness and kinetics of ondansetron as antiemetic remedy after xenon or sevoflurane anesthesia.

4 TRIAL DESIGN

4.1 A specific statement of the primary endpoints and the secondary endpoints, if any, to be measured during the trial.

4.1.1 Primary endpoints:

1. The average depths of hypnosis as assessed by the BIS and the cAAI between skin incision and start of suture.
2. Postoperative nausea as assessed by a verbal rating scale (VRS) ranging between 0 and 10 after anesthesia at 5, 10, 15, 30, 45, 60, and 90 min. At 2, 6 and 24 h after anesthesia the maximum nausea will be rated for the 30-120 min, 2-6 h, and 6-24 h interval.
3. Reduction of VRS nausea immediately at 2, 5, 7.5, 10, 15, 20 and 30 min after rescue treatment administration. Thereafter maximum nausea will be rated at 2, 6 and 24 hours after treatment for the 30-120 min, 2-6 h and 6-24 h interval.

4.1.2 Key secondary endpoint(s):

- Heart rate and blood pressure
- Observer´s assessment of alertness and sedation scales

- Sensitivity and specificity characteristics for both the BIS and the cAAI
- Awareness after anesthesia assessed by the Brice questionnaire at 2 and 24 hours after anesthesia
- Occurrence of postoperative vomiting and the respective time-points will be recorded. Postoperative vomiting is defined as vomiting or retching
- Usage of rescue medication, time and dosage
- Time to discharge from post anesthetic care unit (Aldrete Score ≥ 9 equals the hypothetic discharge time from post anesthetic care unit)

4.2 A description of type/design of trial to be conducted.

Phase IV, prospective, multi-factorial, 3-fold randomized, single blind, parallel group, partly placebo controlled and mono-centric study.

4.3 A description of measures taken to minimize/avoid bias, including:

a) Randomization. A 3-fold randomization will be performed, transacted with a computer generated allocation sequence:
1) The use of xenon or sevoflurane during anesthesia.
2) The use of dexamethasone or placebo for prevention of PONV after xenon or sevoflurane.
3) The use of ondansetron as rescue for the treatment of established PONV after xenon or sevoflurane.

b) Blinding.
1) The use of xenon or sevoflurane will be performed single blinded way, blinded only to the patient, owing to the different MAC- values of the used volatile anesthetics.
2) The use of dexamethasone or placebo will be performed double blinded to both the patient and the physician. The syringes containing the investigatory product or placebo will be provided by the Central Pharmacy (University Hospital Aachen) and will be labeled in a blinded way.
3) The use of ondansetron or placebo will also be performed double blinded to both the patient and the physician. The syringes containing the investigatory product or placebo will be provided by the Central Pharmacy (University Hospital Aachen) and will be labeled in a blinded way.

4.4 A description of the trial treatments and the dosage and dosage regimen of the investigational products. Also include a description of the dosage form, packaging, and labelling of the investigational products.

1. Patients included into the trial will randomly be allocated to either 0.8-1.1 minimum alveolar concentration (MAC) xenon in 30 % oxygen or 0.8-1.1 MAC Sevoflurane (age adapted)/30 % oxygen, depending on the grade of depth of anesthesia required for the current operative stimulus. The MAC is defined and will therefore be applied according to the investigated subject`s age. The duration of the exposition to inhalational anesthetics depends on the duration of the operation and will be terminated after the end of surgery and surgical procedures (i.e. finish of suture, dressing) .
2. All Patients included into the trial have a moderate risk to develop PONV. They will be randomized to a second factor, i.e. a prevention treatment with dexamethasone against PONV versus placebo treatment. The prevention treatment consists of a single dose of 4 mg dexamethasone, applied as i.v. bolus after induction of general anesthesia, or the same volume of NaCl 0.9% as placebo. As soon as a patient has given written confirmed consent to participate in this trial a patient identification number is provided. The Central Pharmacy (University Hospital Aachen; Head of the department: Dr. rer. nat. Albrecht Eisert;

Steinbergweg 20; D-52074 Aachen) will prepare syringes, ready to use according to the randomisation list containing investigational product or placebo and labeled to fulfill the double blinding criteria. The investigational products and placebo will be prepared and stored in accordance with the product information.

3. Patients who experience significant nausea after general anesthesia is terminated i.e. during their stay in the PACU will be randomized to receive a rescue treatment (ondansetron) versus placebo. The rescue treatment consists of a single dose of 4 mg ondansetron applied as i.v. bolus shortly after the occurrence of nausea, or the same volume of NaCl 0.9 % as placebo applied as i.v. bolus. As soon as a patient has given written confirmed consent to participate in this trial a patient identification number is provided. The Central Pharmacy (University Hospital Aachen; Head of the department: Dr. rer. nat. Albrecht Eisert; Steinbergweg 20; D-52074 Aachen) will prepare syringes, ready to use according to the randomisation list containing investigational product or placebo and labeled to fulfill the double blinding criteria. The investigational products and placebo will be prepared and stored in accordance with the product information.

4.5 The expected duration of the subject participation, and a description of the sequence and duration of all trial periods, including follow-up, if any.

The duration of the drug exposition is defined through the time of general anesthesia needed for surgery and the occurrence of post operative nausea. Patients will be monitored closely during the period of general anesthesia including induction and ending, in the post-anesthesia care unit and on the ward by a specially trained investigator up to 24 hours after anesthesia. Patients may be contacted later for other safety or efficacy data.

4.6 A description of ‘stopping rules’ or ‘discontinuation criteria’ for individual subjects, parts of the trial and entire trial.

If during general anesthesia any unexpected serious adverse events not associated with the investigations occur – e.g. severe surgical complications such as mass bleeding or perforation of organs, or severe complications associated with the patients’ individual health conditions such as cardiovascular or respiratory insufficiency or allergic reactions that require CPR or further intensive care treatment, this patient will be taken out of the trial to not interfere with his further therapy.
If during general anesthesia any event occurs that arouses the suspicion of an ongoing Malignant Hyperthermia –e.g. tachycardia/-arrhythmia with no other cause to find, excessive increasing of end-tidal carbon dioxide, increase of body temperature more than 0.5°C over 15 min - the administration of inhalational anesthetics will be immediately stopped and the patient will be taken out of the trial and treated according to the guidelines for treatment of Malignant Hyperthermia.
Any patient can withdraw from the trial without stating reasons at any time. Receiving further rescue treatment on demand or ongoing PONV symptoms is no discontinuation criteria for the trial.
As the investigational products used in this trial are drugs of everyday general anesthesia, which have been observed in numerous studies and everyday anesthetic practice for years, a need to stop parts of or the entire trial out of treatments’ reasons is not expected.

4.7 Accountability procedures for the investigational products, including the placebos.

The investigational product xenon (LENOXe) will be provided by Air Liquide Medical GmbH (Hans-Günther-Sohl-Str.5; D-40235 Düsseldorf). The investigational product sevoflurane (Sevorane®) will be provided by Abbott GmbH & Co. KG (Max-Planck-Ring 2; D-65205 Wiesbaden). Both investigational products (xenon and sevoflurane) will be ordered and distributed through the Central Pharmacy (University Hospital Aachen; Head of the department: Dr. rer. nat. Albrecht Eisert; Steinbergweg 20; D-52074 Aachen). The accountability for the correct use, according to the product information and in consensus with the "Deutschen Gesellschaft für Anästhesiologie und Intensivmedizin" of the above mentioned investigational products remains at the Department of Anesthesiology (University Hospital Aachen; Head of the Department: Univ.-Prof. Dr. med. Rolf Rossaint; Pauwelsstr. 30; D-52074 Aachen).
The investigational product dexamethasone (Fortecortin Inject 4 mg) will be provided by Merck Pharma GmbH (Alsfelder Str. 17; D-64289 Darmstadt) and the investigational product ondansetron (Zofran® i.v. 4mg) will be provided by GlaxoSmithKline GmgH & Co. KG (D-80700 München). Both investigational products (dexamethasone and ondansetron) will be applied double-blind placebo controlled (same volume of NaCl 0.9 %) way. As soon as patient has given written confirmed consent to participate in this trial a patient identification number is provided. The Central Pharmacy (University Hospital Aachen; Head of the department: Dr. rer. nat. Albrecht Eisert; Steinbergweg 20; D-52074 Aachen) will prepare syringes, ready to use according to the randomisation list containing investigational product or placebo and labeled to fulfill the double blinding criteria. The investigational products and placebo will be prepared and stored in accordance with the product information.

4.8 Maintenance of the trial treatment randomization codes and procedures for breaking codes.

The investigators will follow the trial´s randomization procedures. The original randomization code will be kept safe by the sponsor; a copy of the randomization code enveloped for emergency cases will be deposited at the trial site. Un-blinding of the randomization code will only be performed due to a serious adverse event. It is recommended to contact the sponsor before the randomization code is opened.
In case of a serious adverse event with medical necessity of knowing the treatment the investigational subject maintained the investigator or another authorized person may open the enveloped randomization code. Date and reason for opening must be marked on the outer side of the envelope. The application of any investigational product will be immediately stopped, and the sponsor is to be contacted for reasons of withdrawing the investigational subject from the trial. Emergency medication and treatment will be applied due to medical necessity, knowledge and clinical practice.

4.9 The identification of any data to be recorded directly on the CRFs, and to be considered to be source data

For each patient participating in the trial a case report form (CRF) with all information regarding examinations, questioning and additional information through personal impressions will be compiled by the investigator. All required data – e.g. ethnical origin, past medical history, pre-existing drug treatment, measurement of blood pressure/ temperature/pulse/ECG-criteria, monitoring of adverse events - will be directly filled in into the CRF. In case of necessity of acquiring data on separate crude data sheets, the crude data sheets will be considered as source and the data acquired will be immediately transmitted into the CRF. Regarding automatically acquired data: files will be directly transferred into

the CRF and checked manually after transmission, data that does not exist as transferrable file but only in printed form will be submitted into the CRF by hand.

5 SELECTION AND WITHDRAL OF SUBJECTS

5.1 Subject inclusion criteria.

- patients ≥ 18 < 75 years
- ASA physical status I-II
- planned duration of anesthesia ≥ 60 minutes
- Apfel score ≥ 2-3
- elective (laparoscopic) surgery (abdominal, gynecological)
- women: with a highly effective contraception, defined as methods with a pearl index < 1 (i.e. hormonal contraceptives, IUD)

5.2 Subject exclusion criteria.

5.2.1 Contraindications and safety measures as prescribed by product information sheets

- history of hypersensitivity to any used drugs or additive components used for preparation and stabilization of the named drugs in this trial
- history or reasonable suspicion of malignant hyperthermia and/or degenerative neuromuscular disease, in the subject observed or blood relatives
- history of liver function disorders, leucocytosis and unclear fever after usage of halogenated anesthetics.
- any indisposition that may be aggravated by the use of the drugs investigated:
  - liver and/or kidney function disorders
  - severe acute or chronic infectious disease (i.e. viral, bacterial, fungal)
  - elevated intracranial pressure
  - history of gastrointestinal ulcer(s) or inflammatory bowel disease
  - severe metabolic disorders
  - hematoporphyria
  - glaucoma
  - hearing disorders
  - any disease including air-filled closed cavities, such as pneumothorax, ileus
- pregnancy and lactation period

5.2.2 Further exclusion criteria

- subjects under the age of 18 years
- ambulatory surgery
- any disease that is associated with the requirement of a high oxygen yield and/or risk of high oxygen consumption:
  - severe lung and/or airway disease
  - coronary heart disease and/or seriously impaired cardiac function
- severe psychiatric disorder
- refusal of participation
- presumed uncooperativeness or legal incapacity

5.3 Subject withdrawal criteria and procedures specifying:

5.3.1 The following withdrawal criteria are stringent to the investigational subjects

- Severe adverse events that do not permit the trial to be continued.
- Undesirable events that, according to the opinion of the principal investigator, must be considered as hazardous for the physical and psychical health of the individual subject when continuing the trial. The intensity of the undesirable event will be evaluated by the principal investigator.
- Clinically significant measured data, that constitute subject exclusion criteria (see 5.2) and/or indicate harm to the investigational subject's health by medical knowledge.
- Violation of the trial protocol
- Noncompliance
- Withdrawal of consent by the investigational subject
- Difficulties arising from administrational problems

5.3.2 When and how to withdraw subjects from the trial/ investigational product treatment.

Each patient can withdraw from the trial without stating reasons at any time. If any unexpected serious adverse events as mentioned above (see 4.6 and 5.3.1) occur during the time of observation, the subject will be withdrawn from the trial and treated according to his actual physical status (5.3.5). If any of the criteria mentioned as exclusion criteria (5.2) occur in subjects during the course of the trial, those individuals will also be withdrawn from the study and treated according to their needs.
Each decision for preterm withdrawal or exclusion of an investigational subject from the trial will be discussed with the sponsor of the trial and the principal investigator beforehand.

5.3.3 The type and timing of the data to be collected for withdrawn subjects.

The type and timing of safety data to be collected in general see 8.1 and 8.2. Additional data regarding the individual case of the investigational subject withdrawn will be collected according to current medical knowledge and practice.
The reason for withdrawal, the time and specific details of withdrawal of an investigational subject will be marked on a separate sheet (annotation sheet) of the CRF. The investigator is to denominate the principal reason for withdrawal of the investigational subject. All security-relevant data until the subject's withdrawal from the trial will be assessed and documented.

5.3.4 Whether and how subjects are to be replaced.

Subjects breaking of the trial will not be replaced.

5.3.5 The follow-up for subjects withdrawn from investigational product treatment/trial treatment.

All study subjects can withdraw from the study without explanation and if symptomatic, receive a non-study rescue treatment. Patients withdrawn from the investigational product will be kept and treated in the post anesthetic care unit or standard care unit until they meet departmental and hospital standards for discharge from the post anesthetic care unit or standard care unit. Every investigational subject being withdrawn or breaking off the trial will be clearly advised to take part in a final examination.

Subjects withdrawn from the investigational product treatment/trial treatment continue to be patients in the University Hospital Aachen and will be treated by the responsible special clinics. For possible consultation the Department of Anesthesia is available at 24 hours every day (contact address and telephone numbers will be handed out with the patients' information sheet).

6 TREATMENT OF SUBJECTS

6.1 The treatments to be administered, including the names of all products, the doses, the dosing schedules, the routes/modes of administration, and the treatment periods, including the follow-up periods for each investigational product treatment/ trial treatment group/ arm of trial.

1. Patients included into the trial will randomly be allocated to either 0.8-1.1 minimum alveolar concentration (MAC) xenon in 30 % oxygen or 0.8-1.1 MAC sevoflurane (age adapted)/30 % oxygen. The MAC is defined and will therefore be applied according to the investigated subject`s age. Premedication will be performed with midazolam 7.5 mg orally 45 min before induction (standard dose and application form for adults as clinical practice of our department). Anesthesia will be induced in both groups with propofol 2 mg/kg i.v. and remifentanil 0.5 mcg/kg/min by infusion over 60 s. For tracheal intubation non-depolarizing neuromuscular blocking agents can be used (rocuronium 0.6 mg/kg). Both groups will receive remifentanil at a base rate of 0.2 mcg/kg/min. Xenon or sevoflurane can be titrated in the range from 0.8-1.1 MAC according to clinical needs based on the patient's hemodynamic, autonomic and somatic signs. Twenty minutes before the estimated cessation of all surgical procedures 0.05 mg kg^{-1} piritramide for post anesthetic pain management will be administered intravenously, as well as a short infusion of metamizole 15 mg kg^{-1}.

Depth of anesthesia (hypnosis) will be monitored with spontaneous EEG (BIS VISTA, Aspect Medical Systems, Newton, MA) and the mid latency auditory evoked potentials including a monitoring variable indicating the patients hypnotic state calculated from the MLAEP and the electroencephalogram, the composite A-Line ARX Index (cAAI) with the AEP Monitor/2 (Danmeter A/S, Odense, Denmark). Dosing will be conducted according to the current clinical standard without the monitoring, thus the anesthesia provider will be blinded towards both measurements.

2. After induction of anesthesia patients will be randomized to a second factor, i.e. 4 mg dexamethasone or placebo for the prevention of PONV. To avoid potential imbalances, this will be achieved using a factorial design. The application of dexamethasone or placebo will be blinded to the investigator assessing postoperative nausea and vomiting.

3. Patients who experience significant nausea will be randomized to receive either 4 mg ondansetron or placebo and the course of nausea will be assessed for > 32 min. Again, the application of ondansetron or placebo will be blinded to the investigator assessing postoperative nausea and vomiting. If the symptoms of postoperative nausea and vomiting persist for more than 32 min after treatment additional rescue treatment will be offered. Of note, all patients are able to receive further rescue treatment at any time point of the study on demand.

6.2 Medication/treatment permitted and not permitted before and/or during the trial.

There are no other limitations in the medication/treatment before and/or during the trial as mentioned in 1.6.1.

6.3 Procedures for monitoring subject compliance.

Not applicable.

7 ASSESSMENT OF EFFICACY

7.1 Specification of the efficacy parameters.

1) To demonstrate that dexamethasone prevents PONV equally well after xenon or sevoflurane.

2) To determine the onset-time of ondansetron when used as rescue medication for postoperative nausea and vomiting.

7.2 Methods and timing for assessing, recording, and analyzing of efficacy parameters.

1) Postoperative nausea as assessed by a verbal rating scale (VRS) ranging between 0 and 10 after anesthesia at 5, 10, 15, 30, 45, 60, and 90 min. At 2, 6 and 24 h after anesthesia the maximum nausea will be rated for the 30-120 min, 2-6 h, and 6-24 h interval.
2) Reduction of VRS nausea immediately at 2, 5, 7.5, 10, 15, 20 and 30 min after rescue treatment administration. Thereafter maximum nausea will be rated at 2, 6 and 24 hours after treatment for the 30-120 min, 2-6 h and 6-24 h interval.
For each patient participating in the trial a case report form (CRF) with all information regarding examinations, questioning and additional information through personal impressions will be compiled by an investigator. Data that exceeds the defined normal range will be evaluated and marked as ‘clinically significant’ (cs) or ‘not clinically significant’ (ncs) by the investigator. Any data marked as cs will be commentated at this point.

8 ASSESSMENT OF SAFETY

8.1 Specification of safety parameters.

Safety data parameters will include hemodynamic monitoring during anesthesia and in the post anesthetic care unit, monitoring of shivering in the post anesthetic care unit, postoperative organ dysfunction and postoperative laboratory data

8.2 The methods and timing for assessing, recording, and analyzing safety parameters.

Patients will be monitored closely in the post anesthetic care unit and on ward by a specially trained investigator up to 24 hours after anesthesia. All collected safety data will be accurately noted in the CRF. Data that exceeds the defined normal range will be evaluated and marked as ‘clinically significant’ (cs) or ‘not clinically significant’ (ncs) by the investigator. Any data marked as cs will be commentated at this point.

8.3 Procedures for eliciting reports of and for recording and reporting adverse event and intercurrent illnesses.

Undesirable side effects and adverse events, if not spontaneously reported by investigational subjects, will be determined by the investigatory physician through non-suggestive questioning and thoroughly documented with each preset assessment point. Any adverse event will be assessed by the investigator according to chapter 5 *Selection and*

Withdrawal of Subjects and all information concerning the event –i.e. the occurrence, the time of cessation, the grade of severity and/or intensity, any coherence with the applied investigational products, the kind of counteractive measures and its outcome – will be recorded in the CRF.
All investigational subjects that experience undesirable events – no matter if the event is linked to any investigational product or not – are to be monitored until symptoms disappear, any abnormal laboratory value has declined to normal or the observed effects can be sufficiently explained.
All serious adverse events will be reported immediately to the sponsor and will be followed promptly by detailed, written reports. The investigator will also comply with the applicable regulatory requirements related to the reporting of unexpected serious adverse drug reactions to the regulatory authority and the Independent Ethical Committee.

8.4 The type and duration of the follow-up of subjects after adverse events.

If adverse events occur, patients will be kept and treated in the post-anesthetic care unit, standard care unit or if necessary intensive care unit until they meet departmental and hospital standards for discharge from the post-anesthetic care unit, standard care unit or intensive care unit. Study subjects continue to be patients after the study in the University Hospital Aachen and will be treated by the responsible clinical departments. For possible consultation in case of the follow-up of subjects after adverse events and with any other problems concerning the study, the Department of Anesthesia is available at 24 hours every day.

9 STATISTICS

9.1 A description of the methods to be employed, including timing of any planned interim analysis.

After inclusion of 60 patients, at the latest 6 months after inclusion of the first patient (1st November 2008 until 31st March 2009) an interim analysis will be scheduled and a study report will be handed in at the latest end of May 2009, both to the sponsor of the study.

9.2 The number of subjects planned to be enrolled. Reason for choice of sample size, including reflections on the power of the trial and clinical justification.

1. Based on our previous data we expect average BIS values of about 40 (standard deviation SD=10) for xenon and 45 (SD=10) for sevoflurane. A similar comparison will be performed for the MLAEP. In order to account for multiple comparisons alpha/2, i.e. 0.025 will be considered to be statistically significant. Therefore, 100 patients per group will lead to 90 % power (beta-error of 0.10) to detect this difference.
2. We expect the xenon group to have a similar PONV incidence compared to the sevoflurane group. Based on the Apfel score we may expect an incidence of PONV of about 60 % in the sevoflurane/placebo group (since very few patients vomit without nausea, the incidences for nausea and PONV are very similar). A reduction of nausea by dexamethasone of about a third would be considered clinically relevant (I.e. from 60 % to 40 %). To have an alpha error of 0.05 and a beta error of 0.2 (i.e. 80 % power), we will need 107 patients per group.
3. The average incidence of PONV will be about 50% over 24 hours. However, nausea in the PACU will rather be in the range of 20%. Those 50 patients will present with an average nausea score of 5 (SD=4) that an effective dose of ondansetron will reduce on

average to about 2.5 (SD=2). To have 80% (beta-error = 0.20) with an alpha error of 0.05, this will require a total of 52 patients.
In conclusion, a total of 220 patients will be included in this project to meet the demands of all three sample size calculations.

9.3 The level of significance to be used.

For detailed description, see above in 9.2.

9.4 Criteria for the termination of the trial.

There are no statistical criteria for the termination of the trial.

9.5 Procedure for accounting for missing, unused, and spurious data.

Not applicable.

9.6 Procedures for reporting any deviations from the original statistical plan.

Any deviations from the original statistical plan will be described and justified in protocol and in the final report.

9.7 The selection of subjects to be included in the analyses.

All randomized subjects will be included in the analyses.

10 DIRECT ACCESS TO SOURCE DATA/DOCUMENTS

10.1 Sponsor Surveillance

During the time the trial is conducted the sponsor is always permitted to inspect the trial site and observe trial progress, operating procedures, original data, and CRFs.

10.2 Independent Superior Surveillance

The investigator/institution will permit trial-related monitoring, audits, Independent Ethics Committee review, and regulatory inspections providing direct access to source data/documents.

10.3 Reporting of Trial Results

Subsequent to the evaluation of the trial results the investigator will compile a complete trial report in accordance with the principles of ICH GCP to be handed out to the sponsor and to be retained for mandatory time. The trial results will be published in the form of several papers.

11 QUALITY CONTROL AND QUALITY ASSURANCE

11.1 Quality control

To assure the acquisition of accurate, congruent and reliable data and to guarantee the utilization of standard terms and standard operational procedures (SOP), this trial will be conducted according to ICH Topic E6 (R1) Guideline for Good Clinical Practice (CPMP/ICH/135/95) and the applicable regulatory requirements.

11.2 Quality assurance

The following provisions for quality control will be implemented:

- tutorial discussions and training to prepare the investigators for the trials SOPs
- monitoring of the investigational subjects after the criteria mentioned above (4.1) During the time the trial is conducted the sponsor is always permitted to inspect the trial site and survey trial progress, operating procedures, original data, and CRFs.
- alignment of original data and CRFs
- dual submission of data including check on plausibility

12 ETHICS

Consultation for approval of the Independent Ethics Committee has been handed in (EK110/08):
Ethik-Kommission an der Medizinischen Fakultät
Universitätsklinikum
Rheinisch-Westfälische Technische Hochschule Aachen

13 DATA HANDLING AND RECORD KEEPING

For each patient participating in the trial a case report form (CRF) with all information regarding examinations, questioning and additional information through personal impressions will be compiled by the investigator. The CRFs will be filled in with non-soluble ink and the original documents will be handed out to the sponsor as far as the trial report has been completed. Subsequent to the evaluation of the trial results the investigator will compile a complete trial report in accordance with the principles of ICH GCP to be handed out to the sponsor.
The sponsor of this trial – i.e. Air Liquide Santé International – is obliged to retain all essential documents – as lined up in chapters 8.2-8.4, ICH Topic E6 (R1) Guideline for Good Clinical Practice (CPMP/ICH/135/95) – as long as the investigated products are for sale on the open market plus two additional years for legal terms. The final trial report has to be retained up to five years beyond. Documents from a preterm cancelled trial are to be preserved for two years after termination.
The investigator will retain all essential documents – namely as lined up see above - as long as possible due to institutional conditions and premises. The investigator/institution also takes measures to prevent accidental or premature destruction of these documents.

14 FINANCING AND INSURANCE

14.1 Financing of the trial.

The trial is sponsored by Air Liquide Santé International. The costs for the project management, including trial design and preparation, statistical planning, protocol, case report form, data management, biometry and quality assurance are provided by the sponsor and the Department of Anaesthesia (University Hospital Aachen; Head of the Department: Univ.- Prof. Dr. med. Rolf Rossaint), for details please see 17.2. The costs for the technical

equipment and trial drugs needed, ethical vote, case payment, fees for the competent authorities and the insurance fees are covered by Air Liquide Santé International.

14.2 Study Budget

See 17.2

14.2 Insurance of the trial.

If, against one's expectations, damage is caused to somebody's health through this trial, the insurance according §40 Abs.1 Nr. 8 and Abs. 3 AMG has been contracted. The insurance certificate is kept ready by the sponsor and the executor of this study, namely the Department of Anesthesiology, University Hospital Aachen.

AXA Corporate Solutions Niederlassung Deutschland
AXA Corporate Solutions Assurance, Paris
Colonia Allee 10 – 20
D- 51067 Köln
+49 (221) 148105
www.axa-corporatesolutions.com
Insurance number: XDE 0016738LI08A
Agreed sum insured accounts for 5.000.000,00 € with a number of 220 subjects included in the trial and 500.000,00 € per person

15 PUBLICATION POLICY

Any and all publications of the results of the Trial which the Principal Institution and/or the Principal Investigator wish to make shall require prior information from Air Liquide Santé International. In consequence the Principal Investigator and the Principal Institution is entitled to publish investigational results, which accrues during the clinical research project, in a neutral manner. The publication right embodies dissertation-, diploma- and study-thesis. The particular manuscripts will be presented to the Sponsor for inspection, to ensure that no confidential matter will be published. Furthermore, Air Liquide Santé International may exceptionally delay publication or disclosure for a term not exceeding three months with effect from the request in the event that Air Liquide Santé International wishes to seek protection of the information in the publication or paper by industrial property rights.

16 SIGNATURES

Dr. med. Mark Coburn
Principal Investigator
Department of Anesthesia
University Hospital Aachen

Aachen, 20.10.2008

Univ-. Prof. Dr. med. Rolf Rossaint
Head of the Investigator´s Institution
Department of Anesthesia
University Hospital Aachen

Aachen, 20.10.2008

17 ANNEX

17.1 Table of Abbreviations

AEP	Auditory Evoked Potentials
ASA	American Society of Anesthesiologists
BIS	Bispectral Index
cAAI	Composite Auditory Evoked Potential Index
CPMP/ICH/135/95	Note for Guidance on Good Clinical Practice
CRF	Case Report Form
cs	clinically significant
Dr. med.	*Doctor medicinae (lat.)*, MD
Dr. rer. nat.	*Doctor rerum naturarum (lat.)*, PhD
EEG	Electroencephalogram
e.g.	*exempli gratia (lat.)*, for example
GCP	Good Clinical Practice
h	hour(s)
i.e.	*id est (lat.),* that is
i.v.	intra venous
IUD	Intrauterine Device
MAC	Minimum Alveolar Concentration
M.D.	Medical Doctor
mg	milligram
min	minute(s)
MLAEP	Mid Latency Acoustic Evoked Potentials
NaCl	Sodium chloride

ncs	non clinically significant
PACU	Post Anesthesia Care Unit
PhD	Doctor of Philosophy
PONV	Post Operative Nausea and Vomiting
SD	Standard Deviation
Univ-. Prof.	*Universitätsprofessor (ger.)*, university professor
VRS	verbal rating scale

17.2 Additional Information

Study Budget is enclosed in a separate sheet.
